# Postoperative Neurological Deficits Following Regional Anesthesia: A Rare Case of Transient Aphasia and Hemiparesis

**DOI:** 10.7759/cureus.81953

**Published:** 2025-04-09

**Authors:** Regina McPherson, Juan Ramon Santos Rivera, Christine Zickler, Guillermo Izquierdo-Pretel

**Affiliations:** 1 Internal Medicine, Florida International University, Florida, USA; 2 Internal Medicine, Ponce Health Sciences University, Ponce, PRI; 3 Medicine, Florida International University, Herbert Wertheim College of Medicine, Miami, USA; 4 Hospital Medicine, Jackson Memorial Hospital, Miami, USA; 5 Internal Medicine, Florida International University, Herbert Wertheim College of Medicine, Miami, USA

**Keywords:** neurological effects of regional anesthesia, post anesthesia aphasia, post anesthesia hemiparesis, regional anesthesia and chronic pain, transient aphasia

## Abstract

Neurological impairments after anesthesia are fairly common and can oftentimes be serious. A thorough patient evaluation is imperative to discriminate between impairments related to the anesthesia itself and more serious, unrelated complications. Transient motor deficits, aphasia, and postoperative delirium have been reported after general anesthesia and can be related to perioperative stress, exacerbation of previous undiagnosed neurological disorders, and the under-reported psycho-emotional effects of undergoing anesthesia. We present the case of a 57-year-old female who developed expressive aphasia and right-sided weakness following elective ankle arthrodesis performed under monitored anesthesia care (MAC) with regional anesthesia. Medical history was significant for type 2 diabetes mellitus (T2DM), hypertension (HTN), chronic kidney disease (CKD), and Charcot joint. Her presentation sparked initial concern for a cerebrovascular event; however, imaging ruled out stroke and large vessel occlusion. Her neurological deficits were attributed to a rare and under-reported anesthesia-related complication: the psycho-emotional effects of regional anesthesia. The patient demonstrated a gradual recovery of speech and motor function without additional interventions and required inpatient rehabilitation to regain functional independence. This case highlights the importance of prompt evaluation of postoperative neurological symptoms, particularly in patients with significant comorbidities, and underscores the need for increased awareness of rare complications associated with anesthesia.

## Introduction

Early detection of neurological impairments requiring intervention is critical, as timely action can significantly impact patient outcomes, particularly when a cerebrovascular event is suspected [1]. A detailed patient history provides valuable insights into predisposing factors for such events, including pre-existing neurological conditions, cardiovascular disorders, recent medication use, and trauma [2]. A comprehensive neurological examination assessing mental status, cranial nerves, motor and sensory functions, coordination, and reflexes is indispensable for localizing lesions within the nervous system [3]. Key findings, such as unilateral motor and sensory deficits, slurred speech, or aphasia, often suggest stroke, though other etiologies like head injury, brain tumors, infections, and dementia must also be considered [4].

Post-anesthesia neurological complications, while uncommon, present a significant clinical challenge. Among these, post-anesthesia aphasia is particularly concerning, with limited cases reported in the literature. Aphasia, defined as impaired comprehension or production of language due to damage to the brain's language centers, typically affects Broca’s or Wernicke’s areas. Although cerebrovascular accidents are the most common cause, other mechanisms, such as regional cerebral ischemia, microembolic events, and residual neuromuscular blockade, have been implicated in anesthesia-related aphasia [5]. In our case, the use of regional anesthesia with a peripheral nerve block and the absence of radiographic evidence of acute stroke suggested that transient regional cerebral ischemia or anesthetic-related neurotoxicity contributed to the development of aphasia, raising important considerations for anesthetic management in similar patients. This condition may range from mild language impairment to complete loss of linguistic function, affecting components such as semantics, grammar, and phonology [6].

Residual neuromuscular blockade, for instance, can manifest as speaking difficulties and psychomotor retardation, particularly in patients with pre-existing cognitive impairments [7]. Regional anesthesia, widely regarded as safe for intraoperative and postoperative pain management, can occasionally lead to rare neurological complications, most of which are sensory-predominant and transient [8]. Postoperative cognitive impairment following general anesthesia, on the other hand, is a well-known phenomenon, with cases described as early as 1955. There have been several studies since then aimed at investigating whether the type of anesthesia given affects the rates of reported postoperative cognitive impairment [9]. What remains a challenge is distinguishing post-anesthesia aphasia from a cerebrovascular event, particularly in patients with risk factors such as diabetes or hypertension. Given our patient's clinical presentation, understanding the interplay between anesthesia-related mechanisms and predisposing factors is crucial for timely recognition and management. This underscores the necessity of a systematic diagnostic approach to enable early recognition, timely management, and avoidance of unnecessary testing or interventions. A deeper understanding of these rare complications is essential for advancing perioperative care and improving overall patient outcomes.

## Case presentation

**Figure 1:**
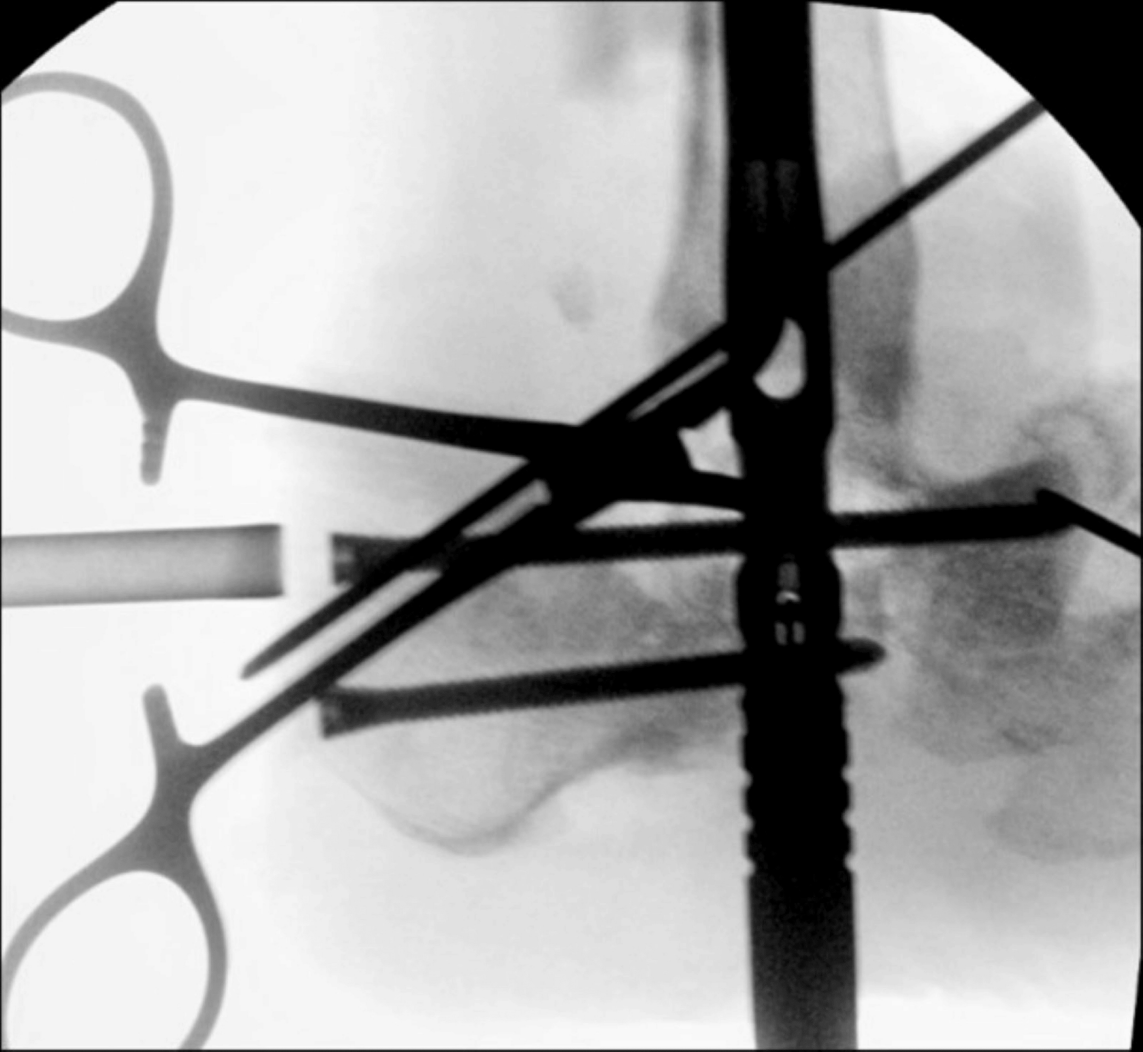
Lateral view of fluoroscopic image demonstrating left ankle arthrodesis and left subtalar joint arthrodesis with the insertion of an intramedullary nail

A 57-year-old female was transferred to the emergency room (ER) from the post-anesthesia care unit (PACU) after presenting with expressive aphasia and right-sided upper and lower extremity weakness. Earlier that day, she underwent elective arthrodesis of the left ankle (Figure 1) under monitored anesthesia care (MAC) with regional anesthesia using a peripheral nerve block. Specifically, 10 mL of 0.5% ropivacaine was administered incrementally over five minutes. Approximately three hours after the surgery, the patient awoke from anesthesia with the noted neurological deficits. Her last known well was prior to surgery.

A stroke alert was initiated, and neurology was consulted. Upon initial evaluation, her blood pressure was 126/78 mmHg, and point-of-care glucose was 169 mg/dL. A non-contrast CT brain showed no acute intracranial hemorrhage; however, she was not a candidate for tenecteplase due to being outside the therapeutic window and her recent surgical procedure. The patient’s medical history included type 2 diabetes mellitus, hypertension, chronic kidney disease, Charcot joint of the ankle, and obesity. At baseline, she was independent in activities of daily living (ADLs) and had no prior history of stroke, alcohol use, or illicit drug use.

**Figure 2:**
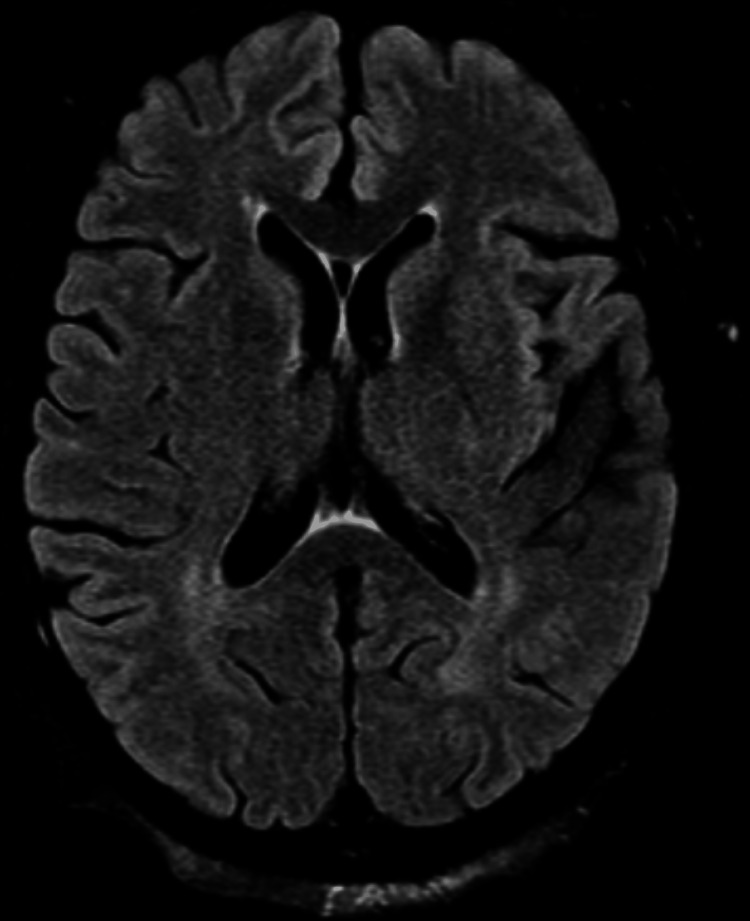
Axial view MRI brain without contrast demonstrating no evidence of acute infarct, midline shift, or hydrocephalus.

In the ER, the patient was awake and alert, following some simple commands. She exhibited stuttering speech without dysarthria, and her thought process remained intact. The cranial nerve examination was normal, including intact pupillary responses. Motor testing revealed significant weakness (1/5) in the right upper and lower extremities, while sensation was intact. Left lower extremity motor strength was not assessed due to pain from the recent surgery. Reflexes were normal, and cerebellar function was intact. Post-surgical laboratory results showed no significant abnormalities (Table 1). Further imaging with CT angiography (CTA) of the head and neck revealed no large vessel occlusions, and mechanical thrombectomy was not indicated. An MRI of the brain showed no acute infarction but did reveal mild chronic microangiopathy (Figure 2).

**Table 1:** Relevant laboratory values post-stroke alert

Laboratory results	Patient results	Reference ranges
White blood cell count	8.7 x10 (3)/mcL	(4.0-10.5) x10 (3)/mcL
Hemoglobin	9.3 g/dL	(11.1-14.6) g/dL
Hematocrit	30.5 %	(33.2-43.4) %
Platelets	218 x10 (3)/mcL	(140-400) x10 (3)/mcL
Glucose	187 mg/dL	74 – 106 mg/dL
Sodium	138 mmol/L	(137-145) mmol/L
Potassium	5.1 mmol/L	(3,6-5.0) mmol/L
Bicarbonate	27 mmol/L	(22-30) mmol/L
Creatinine	1.85 mg/dL	(0.52-1.04) mg/dL
Blood urea nitrogen	42 mg/dL	(7-17) mg/dL
Calcium	8.4 mg/dL	8.4 – 10.2 mg/dL

Over the next few hours, the patient began showing improvement in both speech and motor function. The neurology team concluded that her symptoms were likely related to anesthesia rather than an acute stroke. She was monitored closely in the hospital and assessed daily by physical therapy. She was also evaluated by speech-language pathology (SLP) and occupational therapy (OT). OT recommended interventions consistent with those of physical therapy, while SLP determined that no follow-up was needed. Over several days, she demonstrated gradual improvement in right-sided weakness and stuttering speech. No additional interventions were performed beyond resuming her home medications.

The patient was discharged to an inpatient rehabilitation center for continued physical therapy and strength improvement. After five weeks of rehabilitation, she showed significant recovery: motor strength in the right upper and lower extremities improved to 4/5, and she regained independent ambulation and sit-to-stand capabilities. However, she still required assistance with most ADLs. Upon discharge home, she was prescribed home physical therapy and provided support from a nursing aide and registered nurse to facilitate her continued recovery.

## Discussion

This case underscores the complexity of diagnosing and managing transient neurological symptoms following surgery, highlighting the importance of a systematic evaluation and a multidisciplinary approach. While anesthesia is a cornerstone of modern surgical practice, it carries risks that can extend beyond the immediate postoperative period. This patient presented with expressive aphasia and right-sided weakness postoperatively, prompting a stroke alert and comprehensive evaluation. Transient aphasia and motor deficits after surgery have a broad differential, including residual effects of anesthesia, transient ischemic attacks (TIAs), and psycho-emotional factors. Imaging and laboratory studies ruled out structural brain injury or ischemic stroke. The rarity of anesthesia-related aphasia emphasizes the need for greater awareness and further research to improve diagnostic and management strategies.

Neurological symptoms, such as aphasia and motor deficits, can arise from residual anesthetic effects. Sedatives and regional anesthesia used during monitored anesthesia care can transiently affect the central nervous system (CNS), mimicking neurological deficits. Studies indicate that both regional and general anesthesia can cause transient aphasia or focal neurological symptoms due to reversible alterations in neurotransmitter systems [4,6]. In this case, the onset of symptoms approximately three hours post-surgery aligns with reported cases of delayed neurological symptoms related to anesthesia, which typically resolve spontaneously within hours to days. This highlights the need for meticulous evaluation to differentiate anesthesia-related complications from cerebrovascular events, particularly in patients with significant comorbidities.

Patients with pre-existing vascular risk factors, such as diabetes, hypertension, and chronic kidney disease, may be more susceptible to transient neurological deficits following anesthesia. These conditions can contribute to underlying cerebrovascular disease, which may impair cerebral autoregulation and increase susceptibility to temporary ischemic changes or anesthesia-related neurological effects. Additionally, metabolic derangements, including poor glucose control and renal dysfunction, can influence postoperative neurological status. While the exact mechanism remains unclear, the transient nature of the patient’s symptoms, their resolution without intervention, and the absence of infarction on MRI suggest an anesthesia-related etiology rather than an acute ischemic event. However, given the patient’s multiple vascular risk factors, the possibility of an undetected cerebral infarction cannot be entirely excluded. Future studies should consider more advanced imaging modalities, such as diffusion-weighted imaging (DWI) with finer slices, to rule out small infarcts definitively in similar cases.

The recovery over several weeks, rather than the typical resolution within hours or days seen in transient anesthesia-related effects, may be attributed to the patient’s underlying cerebrovascular disease and microangiopathy, which could have prolonged neurovascular recovery. The patient’s risk factors - diabetes, hypertension, and chronic kidney disease (CKD) - placed her at an elevated risk for cerebrovascular events, including transient ischemic attacks (TIAs) and ischemic strokes. TIAs are characterized by transient neurological deficits that resolve within 24 hours and typically show no evidence of ischemia on imaging [2]. However, while this patient’s presentation initially raised concern for a cerebrovascular event, her clinical course was inconsistent with an acute infarction. Ischemic strokes typically present with persistent deficits, whereas this patient demonstrated significant recovery within days. MRI findings further supported this, as there was no evidence of acute infarction or features suggestive of cytotoxic edema, which would typically be seen as restricted diffusion on DWI. Despite the absence of infarction on imaging, the presence of chronic microangiopathy suggests an underlying vulnerability of the cerebral vasculature, potentially leading to delayed recovery following anesthesia-induced hypoperfusion or transient ischemia. Additionally, the presence of chronic microangiopathy on imaging underscores her underlying vascular risk but does not account for the acute presentation. The transient nature of her deficits, combined with negative imaging findings, strongly suggests an anesthesia-related neurological effect rather than a cerebrovascular event. Nonetheless, it is important to acknowledge that her prolonged recovery over several weeks with rehabilitative therapy is more commonly seen in cases of stroke rather than transient anesthetic effects, which usually resolve within hours to days. This discrepancy raises the need for continued follow-up and consideration of repeat imaging in similar cases to better characterize the etiology of prolonged postoperative neurological deficits.

The gradual improvement over five weeks of rehabilitation further supports the idea that while anesthesia played a role, the patient’s pre-existing microvascular disease likely contributed to a prolonged recovery trajectory. Psycho-emotional responses to surgery and anesthesia, including functional aphasia or neurological symptoms, represent another consideration. Such conditions are usually reversible and not associated with structural abnormalities. In this case, the patient exhibited intact cognition and stuttering speech, with gradual resolution of symptoms over time, suggesting a stress-induced or functional neurological cause for the aphasia [3,5].

Postoperative delirium or cognitive impairment, although a common complication following surgery, was less likely in this case. Delirium is characterized by acute confusion, disorganized speech, fluctuating consciousness, and impaired attention, which can mimic neurological deficits. However, the patient demonstrated preserved cognition, intact thought processes, and the ability to follow simple commands. Her stuttering speech lacked features typically associated with delirium such as disorientation or fluctuating mental status. Furthermore, the rapid improvement of symptoms makes this diagnosis improbable. Correctly distinguishing between delirium and other causes is essential to avoid unnecessary interventions and ensure proper management [10,11].

Metabolic disturbances and infections are also critical differential diagnoses for postoperative neurological symptoms [12]. In this case, mild hyperkalemia and elevated creatinine levels were noted but were not significant enough to explain the patient’s neurological findings. Additionally, laboratory and clinical assessments ruled out infection as a potential cause.

The activation of a stroke alert upon the patient’s presentation illustrates the importance of prioritizing life-threatening conditions in acute scenarios. Prompt recognition and intervention in cases of stroke are crucial to optimizing outcomes [1]. The comprehensive imaging studies, including CT, CTA, and MRI, excluded large vessel hemorrhage, ischemic stroke, and large vessel occlusion. This thorough diagnostic approach underscores the value of a systematic evaluation in avoiding unnecessary treatments while ensuring appropriate management.

Rehabilitation was integral to the patient’s recovery. Physical therapy focused on improving strength and functionality, resulting in significant progress during her five-week inpatient rehabilitation. Despite residual deficits requiring home nursing support, the patient regained partial independence in activities of daily living and achieved functional ambulation. The gradual improvement in her symptoms suggests a favorable prognosis. However, the presence of microangiopathy on MRI necessitates ongoing management, including tight control of blood pressure, blood sugar, and renal function, to mitigate the risk of future cerebrovascular events. Continued rehabilitation should also remain a priority.

This case illustrates the importance of considering a broad differential diagnosis for postoperative neurological symptoms, maintaining a systematic evaluation approach, and emphasizing targeted rehabilitation. The diagnostic workup ruled out critical causes, such as ischemic stroke and infection, ultimately identifying anesthesia-related complications as the likely etiology. Effective management required multidisciplinary collaboration and a focus on rehabilitation, highlighting the need for heightened awareness of rare anesthesia-related neurological complications.

## Conclusions

This case underscores the diagnostic challenges of identifying postoperative neurological symptoms, such as aphasia and motor deficits, which arise following regional anesthesia. It suggests that underlying cerebrovascular disease and chronic microangiopathy may contribute to delayed recovery after regional anesthesia. The patient’s favorable recovery trajectory emphasizes the importance of a multidisciplinary approach, involving collaboration between anesthesiology, neurology, and physical therapy to optimize patient outcomes. This case serves as a valuable educational resource for healthcare providers, demonstrating the need for a systematic diagnostic approach and heightened vigilance in evaluating post-anesthesia complications. A better understanding of this area could refine differential diagnoses, improve risk stratification, and inform preventative strategies for such rare complications in the future.
